# Prevalence of the Absence of Cirrhosis in Subjects with NAFLD-Associated Hepatocellular Carcinoma

**DOI:** 10.3390/jcm10204638

**Published:** 2021-10-09

**Authors:** Marco Castellana, Rossella Donghia, Luisa Lampignano, Fabio Castellana, Roberta Zupo, Rodolfo Sardone, Giovanni De Pergola, Gianluigi Giannelli

**Affiliations:** 1 Unit of Research Methodology, Health Data Sciences and Technology, National Institute of Gastroenterology “Saverio de Bellis”, Research Hospital, Castellana Grotte, 70013 Bari, Italy; rossydonghia@gmail.com (R.D.); luisa.lampignano@irccsdebellis.it (L.L.); fabio.castellana@irccsdebellis.it (F.C.); roberta.zupo@irccsdebellis.it (R.Z.); rodolfo.sardone@irccsdebbellis.it (R.S.); giovanni.depergola@irccsdebellis.it (G.D.P.); 2 Scientific Direction, National Institute of Gastroenterology “Saverio de Bellis”, Research Hospital, Castellana Grotte, 70013 Bari, Italy; gianluigi.giannelli@irccsdebellis.it

**Keywords:** cirrhosis, hepatocellular carcinoma, non-alcoholic fatty liver disease, non-alcoholic steatohepatitis, meta-analysis

## Abstract

Background. Hepatocellular carcinoma (HCC) is most commonly considered as a complication of cirrhosis. However, an increasing number of HCC in subjects with non-alcoholic fatty liver disease (NAFLD) without cirrhosis is being reported. We conducted a meta-analysis to assess the prevalence of the absence of cirrhosis in NAFLD-associated HCC. Methods. Four databases were searched until March 2021 (CRD42021242969). The original articles included were those reporting data on the presence or absence of cirrhosis among at least 50 subjects with NAFLD-associated HCC. The number of subjects with absent cirrhosis in each study was extracted. For statistical pooling of data, a random-effects model was used. Subgroup analyses according to the continent, target condition and reference standard for the diagnosis of cirrhosis were conducted. Results. Thirty studies were included, evaluating 13,371 subjects with NAFLD-associated HCC. The overall prevalence of cases without cirrhosis was 37% (95%CI 28 to 46). A higher prevalence was reported in Asia versus Europe, North America and South America (45, 36, 37 and 22%, respectively) as well as in studies adopting histology only as the reference standard for the diagnosis of cirrhosis versus histology and other modalities (e.g., radiology, endoscopy, biochemistry or overt clinical findings) (53 and 27%, respectively). No difference was found between studies including subjects with non-alcoholic steatohepatitis (NASH) only, versus NAFLD with or without NASH (*p* = 0.385). One in three subjects with NAFLD-associated HCC presented without cirrhosis. This should be reflected in future guidelines and surveillance programs adapted to allow for the early detection of these cancers too.

## 1. Introduction

Non-alcoholic fatty liver disease (NAFLD) is a common disorder with a high prevalence, morbidity and excess mortality rates, that has a major impact on affected subjects, their families and the health-care system. Globally, about one in four subjects is estimated to suffer from this condition, and an even higher frequency is reported among specific populations [1]. In recent years, it has become the leading cause of chronic liver disease and the fastest growing cause of liver transplantation [2]. Indeed, the introduction of new direct-acting antiviral drugs has dramatically improved the outcome of patients affected by hepatitis C virus, and rates in liver transplantation for this indication are declining in several countries [2,3]. By contrast, the prevalence of NAFLD is increasing worldwide and no pharmacological therapy is yet available [4]. 

The natural history of NAFLD is generally characterized by the progression from simple steatosis to inflammation (non-alcoholic steatohepatitis (NASH)) and subsequent fibrosis, cirrhosis and hepatocellular carcinoma (HCC). Subjects with cirrhosis carry the highest risk of adverse clinical outcomes, including end-stage liver disease, esophageal varices and HCC. Therefore, current guidelines recommend that these patients be entered into surveillance programs [5]. Conversely, they do not support routine surveillance for HCC in patients with non-cirrhotic NAFLD, since the individual risk of the progression of these subjects is considered lower [5,6]. However, surveillance programs allow for the detection of HCC at an early stage and an increasing number of patients suffering from NAFLD-associated HCC in the absence of cirrhosis has been reported [7]. It is not clear whether guidelines have accounted for this issue [6]. The aim of the present meta-analysis was to achieve solid information on the prevalence or absence of cirrhosis in subjects with NAFLD-associated HCC.

## 2. Materials and Methods

This meta-analysis was registered in PROSPERO (CRD42021242969) and performed in accordance with the PRISMA Statement [8].

### 2.1. Search Strategy

A six-step search strategy was planned. Firstly, we searched for sentinel studies in PubMed, in the sense of those studies considered fundamental to our systematic review. Secondly, we identified keywords in PubMed. Thirdly, PubMed was searched. Fourthly, CENTRAL, Scopus and Web of Science were searched using the same strategy. Fifthly, studies with available data on the presence or absence of cirrhosis among subjects with NAFLD-associated HCC were selected. Studies meeting the following criteria were excluded: (1) less than 50 subjects; (2) focusing on NAFLD-associated HCC with or without cirrhosis only; (3) reviews, letters, commentaries, abstracts and posters. Lastly, the references of included studies were searched to find additional papers. The last search was performed on 15 March 2021. No language restriction was adopted. Four investigators (M.C., F.C., L.L., R.Z.) independently searched for papers, screened the titles and abstracts of the retrieved articles, reviewed the full texts and selected articles for inclusion. Disagreements were resolved using a consensus-based discussion.

### 2.2. Data Extraction

The following information was extracted independently by the same investigators in a piloted form: (1) general information on the study (author, year of publication, country, study type, study period); (2) target condition (e.g., NAFLD with or without NASH versus NASH only); (3) reference standard for the diagnosis of steatosis; (4) criteria for the diagnosis of NAFLD; (5) reference standard for the diagnosis of HCC; (6) reference standard for the diagnosis of cirrhosis; and (7) numbers of subjects without cirrhosis among those diagnosed with NAFLD-associated HCC. For each selected article, the main paper and Supplementary Data were searched; if data were missing, the authors were contacted via email. Data were cross-checked, and any discrepancy was discussed.

### 2.3. Study Quality Assessment

The risk of bias of included studies was assessed independently by four reviewers (M.C., F.C., L.L., R.Z.) through a quality assessment tool adapted from the National Heart, Lung and Blood Institute [9].

### 2.4. Data Analysis

The characteristics of included studies were summarized, and a meta-analysis of proportion was carried out to obtain the pooled rate with 95% confidence intervals (95%CI) of absent cirrhosis among NAFLD-associated HCC. For statistical pooling of data, a random-effects model was applied. Subgroup analyses were made according to the following variables: (1) continent; (2) target condition; and (3) reference standard for the diagnosis of cirrhosis. Heterogeneity between studies was assessed using I2; a rating of 50% or higher values was considered high heterogeneity. Funnel plots and Egger tests were carried out to evaluate the possible presence of significant publication bias. All analyses were performed per subject using STATA 16.0 (StataCorp software, 2019, Stata Statistical Software, Release 16, College Station, Texas, USA, StataCorp LLC). Significance was set at *p* < 0.05.

## 3. Results

### 3.1. Study Characteristics

**Figure 1:**
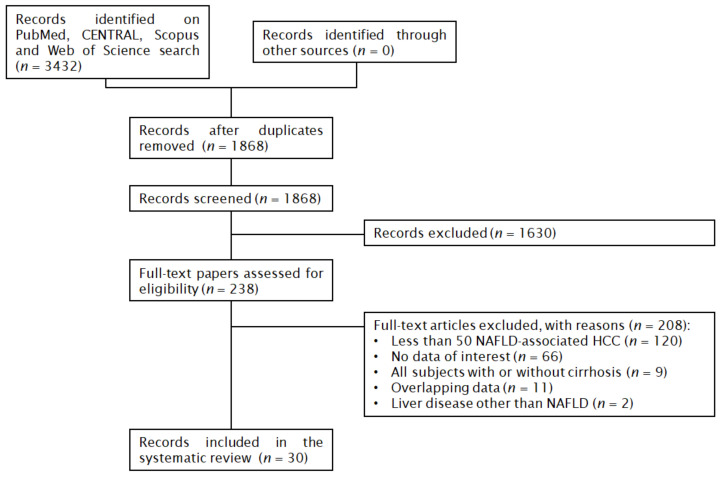
Flow chart of the systematic review.

In total, 3432 papers were found: 969 on PubMed, 137 on CENTRAL, 1429 on Scopus and 897 on Web of Science. After the removal of 1564 duplicates, 1868 articles were analyzed for the title and abstract; 1630 records were excluded (meta-analysis, review, all subjects with or without cirrhosis, less than 50 NAFLD-associated HCC, poster, editorial, letter, outside the scope of the review). The remaining 238 papers were retrieved in full-text and 30 articles were finally included in the meta-analysis (Figure 1) [10,11,12,13,14,15,16,17,18,19,20,21,22,23,24,25,26,27,28,29,30,31,32,33,34,35,36,37,38,39]. No additional studies were retrieved from the references of the included studies.

### 3.2. Qualitative Analysis 

The characteristics of the included articles are summarized in Table 1 [10,11,12,13,14,15,16,17,18,19,20,21,22,23,24,25,26,27,28,29,30,31,32,33,34,35,36,37,38,39]. The studies were published between 2010 and 2021 and had sample sizes ranging from 52 to 5898 patients. Twenty-two studies were retrospective cohorts and six prospective cohorts; the design was not reported in two studies [26,35]. Seven studies were conducted in the United States of America, five in Japan, three in Germany, two in the Republic of Korea, two in the United Kingdom, one in Argentina, one in Australia, one in Brazil, one in China, one in France, one in Italy, one in Singapore, one in Sweden and one in the Netherlands; two studies were multicentric [18,26]. Participants were adult subjects with NAFLD-associated HCC; five studies included subjects with NASH-associated HCC only [11,14,15,21,38]. Details on the diagnostic criteria and reference standards are reported in Table S1. Four studies used health care claims data from large databases [16,22,30,36]. Concerning the remaining studies, the reference standard for the diagnosis of steatosis was generally histology, with or without clinical findings (e.g., metabolic syndrome) or radiology; laboratory data were only adopted in one study [22]. HCC was diagnosed by histology or radiology. Finally, the reference standard for cirrhosis was generally histology with or without radiology, biochemistry, endoscopy or overt clinical findings. Overall, 13,371 subjects with NAFLD-associated HCC were included.

### 3.3. Quantitative Analysis

**Figure 2:**
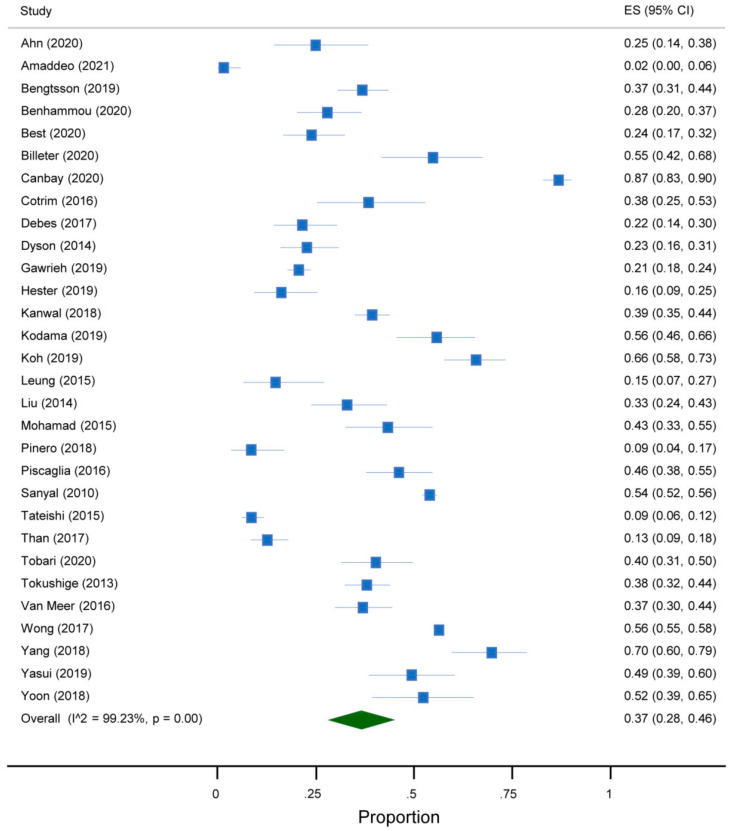
Forest plot of the absence of cirrhosis in subjects with NAFLD-associated HCC.

**Figure 3:**
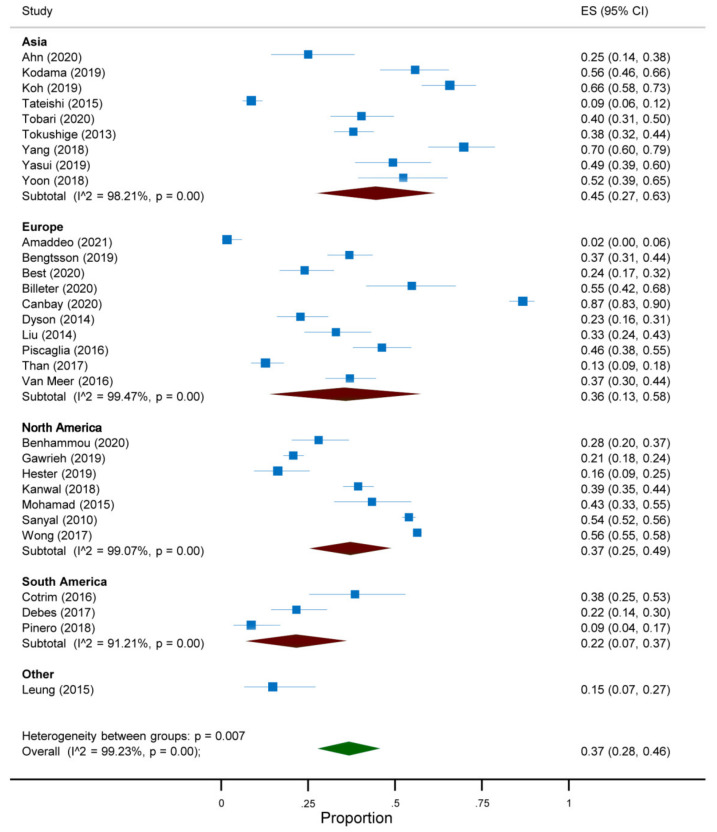
Forest plot of the absence of cirrhosis in subjects with NAFLD-associated HCC according to the continent.

**Figure 4:**
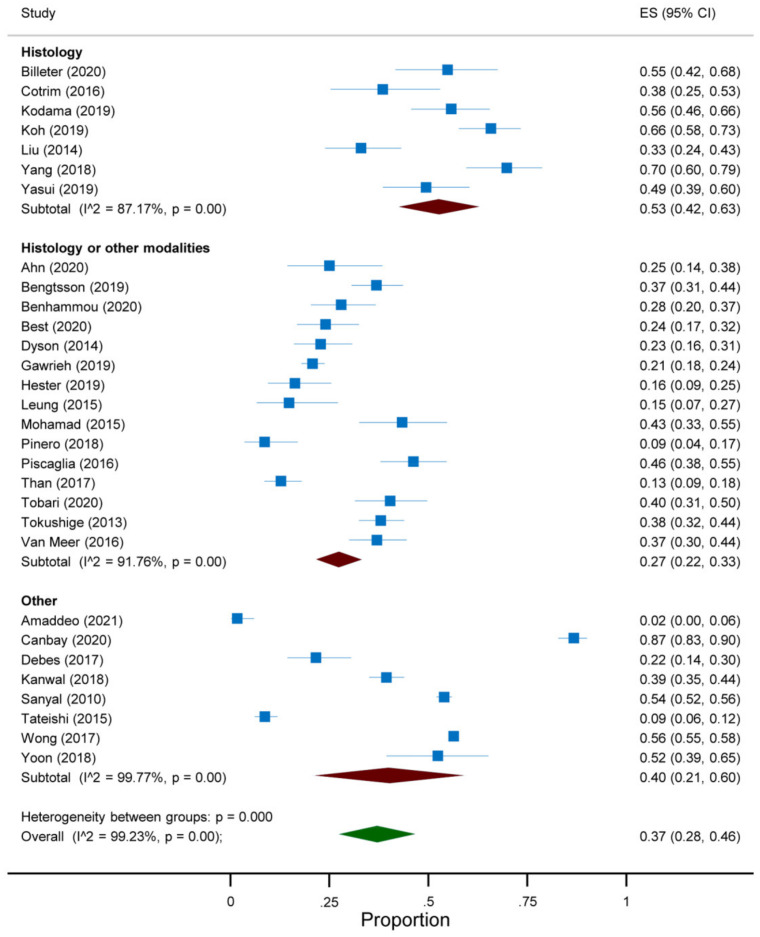
Forest plot of the absence of cirrhosis in subjects with NAFLD-associated HCC according to the reference standard for the diagnosis of cirrhosis.

**Figure 5:**
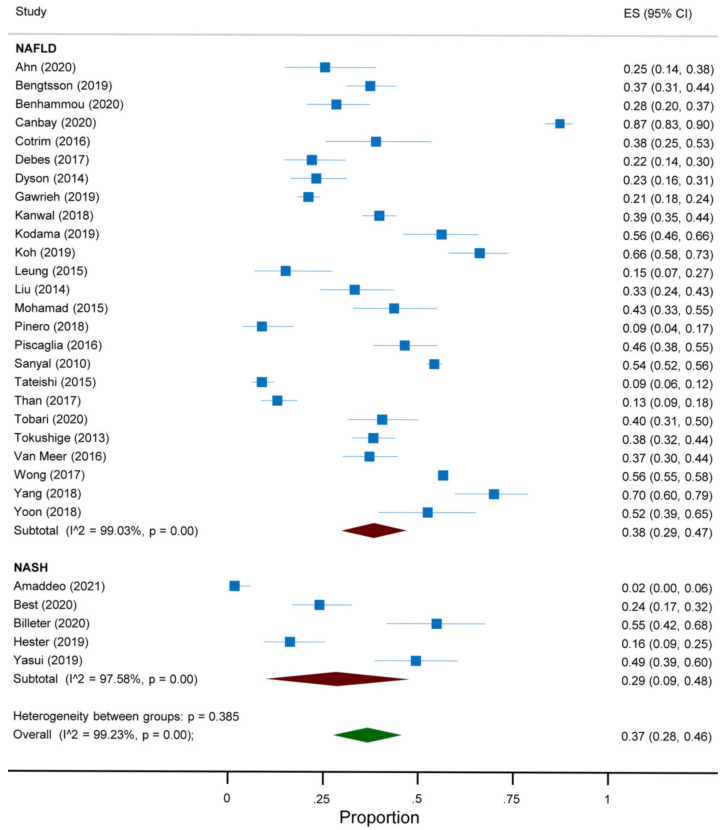
Forest plot of the absence of cirrhosis in subjects with NAFLD-associated HCC according to the target condition.

The overall prevalence of absent cirrhosis among NAFLD-associated HCC in all the articles included in the meta-analysis was 37% (95%CI 28 to 46) (Figure 2). This prevalence differed according to the continent and the reference standard for the diagnosis of cirrhosis. Indeed, a prevalence of 45% (95%CI 27 to 63) was reported among studies conducted in Asia, compared to 37% (95%CI 25 to 49) in North America, 36% (95%CI 13 to 58) in Europe and 22% (95%CI 7 to 37) in South America (Figure 3). Additionally, a prevalence of 53% (95%CI 42 to 63) was reported in studies adopting histology only as the reference standard for the diagnosis of cirrhosis, compared to 27% (95%CI 22 to 33) in those adopting histology and other modalities and 40% (95%CI 21 to 60) in the remaining studies (Figure 4). As regards the target condition, no difference was found among the studies including subjects with NASH only versus NAFLD with or without NASH (29% vs. 38%, *p* = 0.385) (Figure 5). A high heterogeneity was found, with no evidence of publication bias (Figure S1).

### 3.4. Study Quality Assessment

The risk of bias in the included studies is shown in Table S2. Overall, the objective of the studies was clearly stated, the study population was clearly identified, all the subjects were recruited from the same population, and the inclusion and exclusion criteria were consistently implemented. No sample size justification was provided in any of the studies, and it was unclear whether the outcome assessors were blinded. The only exception to the statements above were two studies with incomplete data on the study population [17,26], four studies with incomplete data on the criteria for the diagnosis of NAFLD/NASH [11,13,18,26] and three studies with incomplete data on the assessment of cirrhosis [11,18,31]. Finally, one study only reported a blinded assessment of the sections of non-tumor liver tissues [38].

## 4. Discussion

The aim of this meta-analysis was to identify the best available evidence on the prevalence of the absence of cirrhosis in subjects with NAFLD-associated HCC. Thirty studies were found, evaluating 13,371 subjects with NAFLD- or NASH-associated HCC. The pooled prevalence of non-cirrhotic liver in these articles was 37%, with heterogeneity among the studies. To our knowledge, this is the largest meta-analysis on the topic. An extensive database search was performed without time or language restrictions, and inclusion criteria were defined prior to the database search. 

Traditionally, the natural history of NAFLD has been considered as a continuum from simple steatosis to inflammation, fibrosis, cirrhosis and its complications, including HCC [40]. However, evidence accumulating in recent years has shown that the pathogenesis of NAFLD-associated HCC may differ from this pathway in some subjects. In particular, a meta-analysis published by Stine et al. showed two-fold higher odds of developing HCC among subjects with non-cirrhotic NASH compared to subjects with other liver disease etiologies (OR 2.61, 95% CI 1.27 to 5.35, *p* = 0.009) [41]. In line with this result, we found that about one in three subjects with NAFLD-associated HCC had no cirrhosis at the cancer diagnosis. The prevalence appeared to be higher in Asia versus Europe, North America and South America, as well as in studies adopting histology only as the reference standard for the diagnosis of cirrhosis compared to other modalities. No difference was found among the studies including subjects with NAFLD with or without NASH compared to NASH only. Ethnicity may be the basis for the geographical finding; indeed, it is common knowledge that Asians are characterized by a higher prevalence of NAFLD and a more severe inflammation at biopsy [42]. Additionally, a higher prevalence of previous or occult HBV infection and carriage of the PNPLA3 polymorphism, both associated with an increased risk of HCC, has been reported in these subjects [43]. Concerning the reference standard for the diagnosis of cirrhosis, the performance of imaging is generally adequate and the same holds true for the other modalities (e.g., evidence of portal hypertension) [44]. Moreover, even if histology is generally considered the gold standard, it may be contraindicated in some subjects (e.g., patients with hepatic decompensation would unlikely have histology and, therefore, be underrepresented in such cohorts) and/or associated with sampling variability/inadequate biopsy samples leading to discordant results. Therefore, our finding of a lower prevalence of non-cirrhotic NAFLD-associated HCC in subjects whose liver was assessed by histology, radiology, endoscopy, biochemistry or overt clinical findings as compared to histology only, should not be interpreted as a different diagnostic accuracy of adopted methods. Rather, it may be viewed as a consequence of the different study design leading to a different selection bias, suggesting that the former studies provide a more reliable estimate. Finally, patients with end-stage liver disease might have burnt-out NASH, and the histologic features of necroinflammation might no longer be apparent; the absence of NASH at the time of HCC diagnosis does not mean that the patient has never had NASH before. Therefore, our findings on differences in the background liver disease should critically be interpreted.

Our data raise the question whether all subjects with NAFLD/NASH should be systematically surveyed for HCC. On one hand, we should aim at the early diagnosis of all HCC, given the clinical implications of those lesions discovered at more advanced stages, as is currently occurring for NAFLD-associated HCC without cirrhosis [45]. Indeed, compared to those with cirrhotic NAFLD-associated HCC, subjects without cirrhosis were more likely to present with a single nodule characterized by a lower liver histological activity, despite a larger tumor size, and they underwent liver transplantation or received loco-regional therapy less frequently, rather resection was more frequently performed, leading to a similar survival [12,23,25,27]. On the other hand, current adherence to surveillance programs among subjects with cirrhosis is poor and the performance of ultrasound in detecting HCC is suboptimal, especially in those subjects with more severe forms of obesity [46,47]. Additionally, given the high prevalence of NAFLD, the annual incidence of HCC is likely to be far from the 1.5% threshold recommended by the AASLD to justify the screening [48]. 

Research is ongoing to further stratify the risk of HCC among non-cirrhotic subjects and define subgroups of patients at higher risk. One study found the risk of HCC to be higher in subjects aged > 55 years with elevated ALT [49]. Another study found that bearing the PNPLA3 rs738409 C > G polymorphism was a risk factor for NAFLD-associated HCC, regardless of the background liver disease [50]. A meta-analysis found an annual incidence of HCC of 5.29 per 1000 person-years among subjects with NASH compared to 0.44 per 1000 person-years among subjects with NAFLD [1]. According to a recent clinical practice update, all subjects with advanced fibrosis defined according to two noninvasive testing modalities should be considered for HCC screening [45]. In conclusion, while routine surveillance for HCC in all subject patients with non-cirrhotic NAFLD cannot be recommended and the strategy to identify those subjects who might best benefit from HCC surveillance is still to be identified, specific recommendations in this field are warranted. Findings from new approaches to data analysis (e.g., artificial intelligence) will hopefully improve our understanding of this issue [51].

In July 2018, a meta-analysis was published by Stine et al. on a similar topic [41]. The prevalence of non-cirrhotic NASH-associated HCC was 38%. This result suffered from some limitations, particularly, (a) it was based on only seven studies; (b) subjects with bridging fibrosis and cirrhosis from Reddy et al. were both classified as cirrhotic [52]; (c) subjects with NAFLD were included, even if the primary outcome was to assess the pooled risk of HCC in patients with NASH [31,34]; (d) data extracted from Tateishi et al. differed from those reported in the original study [31]; and (e) data were summed, not pooled into a meta-analysis. Five studies were excluded from our analysis on the basis of our protocol. The results of our meta-analysis were based on 28 additional articles and on separate analyses conducted according to the continent, target condition and reference standard for the diagnosis of cirrhosis. This resulted in a more objective and accurate interpretation of the available evidence.

The limitations of the present paper should be discussed. The first limitation relates to the design of included studies: a retrospective review of subjects with NAFLD-associated HCC was performed in most of them, and this introduced a significant selection bias. We selected only those studies in which an arbitrary number of at least 50 subjects with NAFLD-associated HCC were included, and this is a second limitation. However, the prevalence of non-cirrhotic NAFLD-associated HCC is expected to be lower compared to cirrhotic HCC; therefore, only studies with an adequate sample size could be deemed sufficiently powered to reliably determine their frequency. Lastly, details on the degree of steatosis, the presence of NASH and the fibrosis stage among subjects without cirrhosis were not systematically reported. Further studies are needed to assess the characteristics of the background liver disease and to assess the long-term implications of different background liver diseases and strategies to diagnose it on HCC-related outcomes. 

## 5. Conclusions

The high prevalence of NAFLD and its projections in the next year pose an important clinical challenge. On one hand, efforts should be made to reduce the number of new cases, thus attenuating the burden of this disorder. On the other hand, strategies should be defined to diagnose complications at the earliest stages with the aim of prolonging survival. The present meta-analysis found that about one in three subjects with NAFLD-associated HCC presented without cirrhosis at diagnosis. Given that to date no screening is recommended in these subjects, our results raise the question as to whether a relevant number of affected subjects is neglected during clinical practice. Further studies investigating this latter issue seem to be urgently needed.

## Figures and Tables

**Table 1:** Characteristics of included studies.

First Author, Year	Country	Study Design	Years	Target Condition ^a^	Participants (*n*)
Ahn, 2020 [10]	Republic of Korea	RCS	2000–2016	NAFLD	56
Amaddeo, 2021 [11]	France	RCS	2019–2020	NASH	119
Bengtsson, 2019 [12]	Sweden	RCS	2004–2017	NAFLD	225
Benhammou, 2020 [13]	USA	RCS	2000–2016	NAFLD	125
Best, 2020 [14] ^b^	Germany	RCS	2001–2016	NASH	125
Billeter, 2020 [15]	Germany	PCS	2001–2017	NASH	62
Canbay, 2020 [16]	Germany	RCS	2011–2016	NAFLD	363
Cotrim, 2016 [17]	Brazil	RCS	-	NAFLD	52
Debes, 2017 [18]	Argentina, Brazil, Colombia, Ecuador, Peru, Uruguay	RCS	2005–2015	NAFLD	111
Dyson, 2014 [19]	United Kingdom	PCS	2000–2010	NAFLD	136
Gawrieh, 2019 [20]	USA	RCS	2000–2014	NAFLD	767
Hester, 2019 [21]	USA	RCS	2008–2016	NASH	92
Kanwal, 2018 [22]	USA	RCS	2003–2011	NAFLD	490
Kodama, 2019 [23]	Japan	RCS	2000–2016	NAFLD	104
Koh, 2019 [24]	Singapore	PCS	2000–2015	NAFLD	152
Leung, 2015 [25]	Australia	RCS	2000–2012	NAFLD	54
Liu, 2014 [26]	Switzerland, United Kingdom	-	-	NAFLD	100
Mohamad, 2015 [27]	USA	RCS	2003–2012	NAFLD	83
Pinero, 2018 [28]	Argentina	PCS	2009–2016	NAFLD	81
Piscaglia, 2016 [29]	Italy	PCS	2010–2012	NAFLD	145
Sanyal, 2010 [30]	USA	RCS	2002–2008	NAFLD	2578
Tateishi, 2015 [31]	Japan	RCS	1991–2010	NAFLD	403
Than, 2017 [32]	United Kingdom	RCS	2000–2014	NAFLD	212
Tobari, 2020 [33]	Japan	PCS	1991–2018	NAFLD	119
Tokushige, 2013 [34]	Japan	RCS	2006–2009	NAFLD	292
van Meer, 2016 [35]	the Netherlands	-	2005–2012	NAFLD	181
Wong, 2017 [36]	USA	RCS	1991–2011	NAFLD	5898
Yang, 2018 [37]	China	RCS	2003–2014	NAFLD	96
Yasui, 2019 [38]	Japan	RCS	1993–2010	NASH	87
Yoon, 2018 [39]	Republic of Korea	RCS	2005–2015	NAFLD	63

^a^ Studies including subjects with NAFLD- with or without NASH-associated HCC were classified as “NAFLD”, while studies including subjects with NASH-associated HCC only were classified as “NASH”; ^b^ in Best, 2020 [14] data from the Japanese cohort were not extracted due to overlap with Tateishi, 2015 [31]; NAFLD, non-alcoholic fatty liver disease; NASH, non-alcoholic steatohepatitis; PCS, prospective cohort study; RCS, retrospective cohort study; -, not reported.

## Data Availability

Data are available from the corresponding author on a reasonable request.

## Supplementary Materials

The following are available online at https://www.mdpi.com/article/10.3390/jcm10204638/s1, Figure S1: Funnel plot of meta-analysis on the absence of cirrhosis in subjects with NAFLD-associated HCC, Table S1: Criteria for the diagnosis of NAFLD/NASH, HCC and cirrhosis, Table S2: Risk of bias of included studies.
