# Supplementary material for: Prevalence of the Absence of Cirrhosis in Subjects with NAFLD-Associated Hepatocellular Carcinoma
Source: J Clin Med. 2021 Oct 9;10(20):4638. doi: 10.3390/jcm10204638 (PMC8539355; doi:10.3390/jcm10204638)

Figure S1. Funnel plot of meta-analysis on the absence of cirrhosis in subjects with NAFLD-associated HCC.

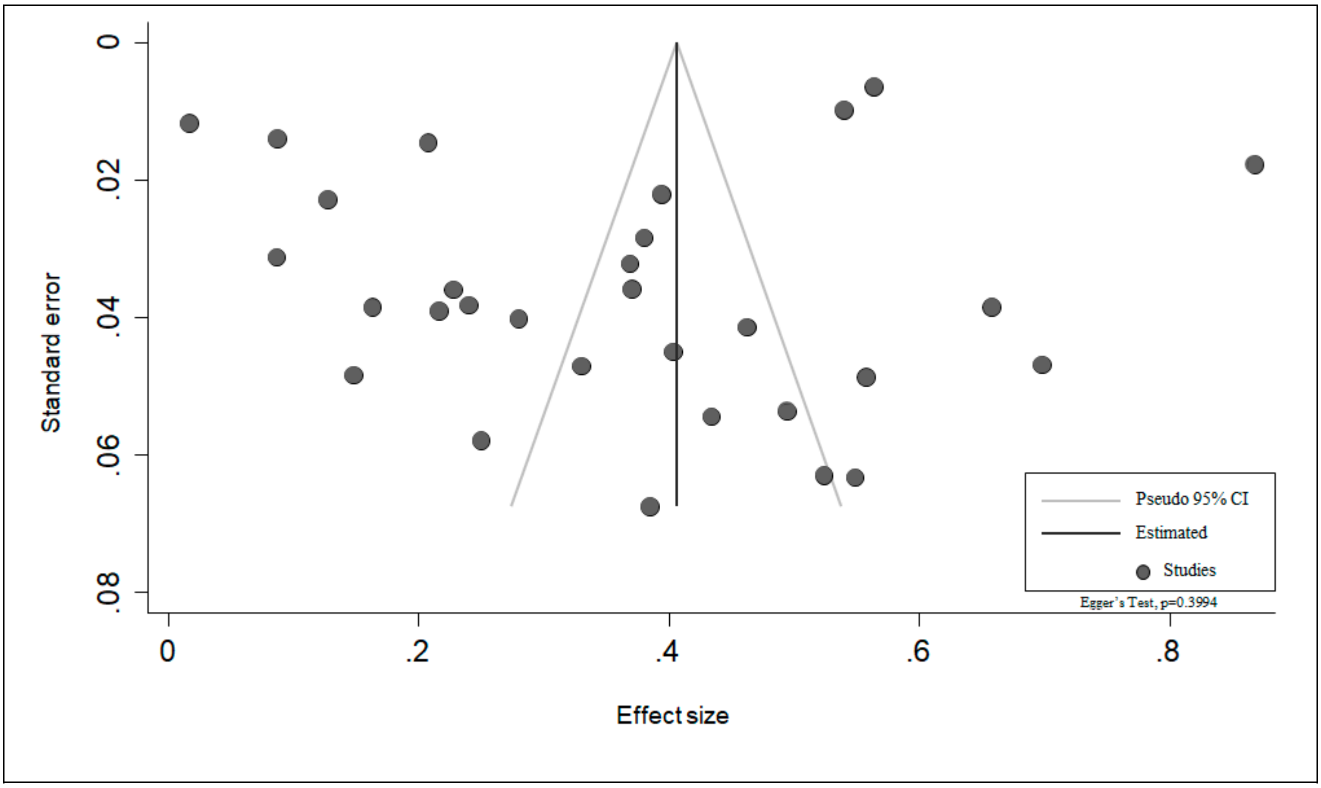

Supplement: Supplementary file 1 [file jcm-10-04638-s001.zip › Figure S1.pdf]
