# Supplementary material for: Prevalence of the Absence of Cirrhosis in Subjects with NAFLD-Associated Hepatocellular Carcinoma
Source: J Clin Med. 2021 Oct 9;10(20):4638. doi: 10.3390/jcm10204638 (PMC8539355; doi:10.3390/jcm10204638)
Supplement: Supplementary file 1 [file jcm-10-04638-s001.zip › Table S1.pdf]

**Table S1. Criteria for the diagnosis of NAFLD/NASH, HCC and cirrhosis.**

| First Author, year | Target condition [a] | Diagnostic criteria for steatosis | Diagnostic criteria for NAFLD | Reference standard for HCC | Reference standard for cirrhosis |
|---|---|---|---|---|---|
| Ahn, 2020 [10] | NAFLD | histology or radiology (US, CT) | HBV, HCV, alcohol, medications, hereditary liver disorders | histology or radiology according to 2018 AASLD | histology, radiology, biochemistry, endoscopy |
| Amaddeo, 2021 [11] | NASH | - | - | histology or radiology according to 2018 EASL | - |
| Bengtsson, 2019 [12] | NAFLD | histology or radiology, BMI≥25 kg/m2 and T2DM, or BMI ≥ 30 kg/m2 | HBV, HCV, alcohol, autoimmune liver disease, hemochromatosis, alpha-1 antitrypsin deficiency and other possible causes of liver disease | histology or radiology (US, MRI, CT) according to 2018 EASL | histology, radiology, endoscopy |
| Benhammou, 2020 [13] | NAFLD | - | HBV, HCV, alcohol, autoimmune liver disease, alpha-1 anti-trypsin, Wilson disease and hemochromatosis | histology or radiology (MRI, CT) according to 2018 EASL | histology, radiology, biochemistry, endoscopy |
| Best, 2020 [14] [b] | NASH | histology or metabolic syndrome | HBV, HCV, alcohol, other possible causes of liver disease | histology or radiology (MRI, CT) according to 2012 EASL | histology or by overt clinical findings as portal hypertension in known chronic liver diseases |
| Billeter, 2020 [15] | NASH | histology | HBV, HCV, alcohol, hemochromatosis, Wilson disease, or other rare diseases | histology | histology |
| Canbay, 2020 [16] | NAFLD | diagnosis-specific claims | HAV, HBV, HCV, HDV, HEV, HIV, alcohol, autoimmune liver disease, toxic liver disease, Wilson disease, Gaucher disease, lysosomal acid lipase deficiency, hemochromatosis | diagnosis-specific claims | diagnosis-specific claims |
| Cotrim, 2016 [17] | NAFLD | risk factors for NAFLD or metabolic syndrome | HBV, HCV, alcohol, hemocromatosis, and autoimmune liver disease | histology | histology |
| Debes, 2017 [18] | NAFLD | - | HCV, HBV, alcohol, hemochromatosis, autoimmune liver disease, schistosomiasis, medications, and vascular forma | histology or radiology according to 2012 AASLD, 2012 EASL-EORTC or 2014 LAASL | - |
| Dyson, 2014 [19] | NAFLD | histology or radiology | negative liver screen and alcohol intake | histology or radiology (MRI, CT) | histology or radiology |
| Gawrieh, 2019 [20] | NAFLD | clinical, histology or radiology | HBV, HCV, alcohol, autoimmune liver disease, haemochromatosis and alpha-1-antitrypsin deficiency | histology or radiology according to 2018 AASLD | histology, radiology, clinical, biochemistry |
| Hester, 2019 [21] | NASH | histology or radiology | HBV, HCV, alcohol, and other liver diseases | histology or radiology according to 2018 AASLD | histology, radiology, clinical, biochemistry |
| Kanwal, 2018 [22] | NAFLD | 2 or more elevated ALT values and more than 6 months apart | HBV, HCV, alcohol, autoimmune liver disease, hemochromatosis, or a-1 antitrypsin disease | diagnosis-specific claims | diagnosis-specific claims |
| Kodama, 2019 [23] | NAFLD | histology | HBV, HCV, alcohol, autoimmune liver disease, medications, biliary obstruction, Wilson disease and hemochromatosis | histology or radiology (US, MRI, CT, selective hepatic arteriography) | histology |
| Koh, 2019 [24] | NAFLD | histology | HBV, HCV, alcohol, autoimmune liver disease | histology | histology |

| First Author, year | Target condition [a] | Diagnostic criteria for steatosis | Diagnostic criteria for NAFLD | Reference standard for HCC | Reference standard for cirrhosis |
|---|---|---|---|---|---|
| Leung, 2015 [25] | NAFLD | histology or radiology | HBV, HCV, alcohol, Wilson disease, haemochromatosis, autoimmune liver disease, alpha1-antitrypsin deficiency, cystic fibrosis, other chronic biliary tract diseases and other hepatic malignancies | histology or radiology according to 2011 AASLD | histology or radiology |
| Liu, 2014 [26] | NAFLD | histology or radiology | - | histology or radiology according to 2012 EASL-EORTC | histology |
| Mohamad, 2015 [27] | NAFLD | histology or radiology | alcohol or confounding liver diseases | histology or radiology according to 2011 AASLD | histology or radiology |
| Pinero, 2018 [28] | NAFLD | histology or radiology (US) | HBV, HCV, alcohol, cryptogenic cirrhosis (CC), autoimmune liver disease, hemochromatosis, Wilson disease, toxic liver disease | histology or radiology according to 2012 EASL-EORTC | histology, radiology, biochemistry, endoscopy |
| Piscaglia, 2016 [29] | NAFLD | radiology (US) | alcohol, other liver diseases | histology or radiology according to 2012 EASL-EORTC | histology, radiology, biochemistry |
| Sanyal, 2010 [30] | NAFLD | diagnosis-specific claims | diagnosis-specific claims | diagnosis-specific claims | diagnosis-specific claims |
| Tateishi, 2015 [31] | NAFLD | histology or radiology | HBV, HCV, alcohol, autoimmune liver disease, Budd-Chiari syndrome, hemochromatosis, Wilson disease, and other liver diseases | histology or radiology (MRI, CT, angiography) | - |
| Than, 2017 [32] | NAFLD | histology or radiology | HCV, alcohol | radiology (US, MRI) | histology or radiology |
| Tobari, 2020 [33] | NAFLD | histology or radiology | HBV, HCV, alcohol and other liver diseases | histology or radiology (US, MRI, CT, selective hepatic arteriography | histology, radiology, endoscopy |
| Tokushige, 2013 [34] | NAFLD | histology or radiology | HBV, HCV, alcohol and other liver diseases | histology or radiology | histology, radiology, biochemistry, endoscopy |
| van Meer, 2016 [35] | NAFLD | histology or metabolic syndrome | HBV, HCV, alcohol, hemochromatosis and other liver diseases | histology or radiology according to 2011 AASLD | histology, radiology, biochemistry |
| Wong, 2017 [36] | NAFLD | diagnosis-specific claims | diagnosis-specific claims | diagnosis-specific claims | diagnosis-specific claims |
| Yang, 2018 [37] | NAFLD | radiology or metabolic syndrome | HBV, HCV, HEV, alcohol, medications, autoimmune liver diseases, Wilson disease, alpha-1-antitrypsin deficiency, hemochromatosis, and Pompe's syndrome | histology | histology |
| Yasui, 2019 [38] | NASH | histology | HBV, HCV, alcohol, autoimmune liver diseases, Wilson disease, or hemochromatosis, medications, gastrointestinal bypass surgery | histology or radiology according to 2005 AASLD | histology |
| Yoon, 2018 [39] | NAFLD | histology or radiology | HBV, HCV, alcohol, medications or hereditary disorders | histology or radiology according to 2011 AASLD | radiology or clinical |
