# Supplementary material for: Prevalence of the Absence of Cirrhosis in Subjects with NAFLD-Associated Hepatocellular Carcinoma
Source: J Clin Med. 2021 Oct 9;10(20):4638. doi: 10.3390/jcm10204638 (PMC8539355; doi:10.3390/jcm10204638)
Supplement: Supplementary file 1 [file jcm-10-04638-s001.zip › Table S2.pdf]

Table S2. Risk of bias of included studies.

First Author, year	1	2	3	4	5	6	7	8	Total
Ahn, 2020 [10]	Y	Y	Y	N	Y	Y	Y	-	6/8
Amaddeo, 2021 [11]	Y	Y	Y	N	Y	N	N	-	4/8
Bengtsson, 2019 [12]	Y	Y	Y	N	Y	Y	Y	-	6/8
Benhammou, 2020 [13]	Y	Y	Y	N	Y	N	Y	-	5/8
Best, 2020 [14] [b]	Y	Y	Y	N	Y	Y	Y	-	6/8
Billeter, 2020 [15]	Y	Y	Y	N	Y	Y	Y	-	6/8
Canbay, 2020 [16]	Y	Y	Y	N	Y	Y	Y	-	6/8
Cotrim, 2016 [17]	Y	N	-	N	Y	Y	Y	-	4/8
Debes, 2017 [18]	Y	Y	Y	N	Y	N	N	-	4/8
Dyson, 2014 [19]	Y	Y	Y	N	Y	Y	Y	-	6/8
Gawrieh, 2019 [20]	Y	Y	Y	N	Y	Y	Y	-	6/8
Hester, 2019 [21]	Y	Y	Y	N	Y	Y	Y	-	5/8
Kanwal, 2018 [22]	Y	Y	Y	N	Y	Y	Y	-	6/8
Kodama, 2019 [23]	Y	Y	Y	N	Y	Y	Y	-	6/8
Koh, 2019 [24]	Y	Y	Y	N	Y	Y	Y	-	6/8
Leung, 2015 [25]	Y	Y	Y	N	Y	Y	Y	-	6/8
Liu, 2014 [26]	Y	N	-	N	Y	N	Y	-	3/8
Mohamad, 2015 [27]	Y	Y	Y	N	Y	Y	Y	-	6/8
Pinero, 2018 [28]	Y	Y	Y	N	Y	Y	Y	-	6/8
Piscaglia, 2016 [29]	Y	Y	Y	N	Y	Y	Y	-	6/8
Sanyal, 2010 [30]	Y	Y	Y	N	Y	Y	Y	-	6/8
Tateishi, 2015 [31]	Y	Y	Y	N	Y	Y	N	-	5/8
Than, 2017 [32]	Y	Y	Y	N	Y	Y	Y	-	6/8
Tobari, 2020 [33]	Y	Y	Y	N	Y	Y	Y	-	6/8
Tokushige, 2013 [34]	Y	Y	Y	N	Y	Y	Y	-	6/8
van Meer, 2016 [35]	Y	Y	Y	N	Y	Y	Y	-	6/8
Wong, 2017 [36]	Y	Y	Y	N	Y	Y	Y	-	6/8
Yang, 2018 [37]	Y	Y	Y	N	Y	Y	Y	-	6/8
Yasui, 2019 [38]	Y	Y	Y	N	Y	Y	Y	Y	7/8
Yoon, 2018 [39]	Y	Y	Y	N	Y	Y	Y	-	6/8

1. Was the research question or objective in this paper clearly stated?

2. Was the study population clearly specified and defined?

3. Were all the subjects selected or recruited from the same or similar populations (including the same time period)?

4. Was a sample size justification, power description, or variance and effect estimates provided?

5. Were the definitions, inclusion and exclusion criteria, algorithms or processes used to identify or select subjects and controls valid, reliable, and implemented consistently across all study participants?

6. Were the exposure measures (independent variables) clearly defined, valid, reliable, and implemented consistently across all study participants?

7. Were the outcome measures (dependent variables) clearly defined, valid, reliable, and implemented consistently across all study participants?

8. Were the outcome assessors blinded to the exposure status of participants?
